# Book Reviews

**Published:** 1802-09-01

**Authors:** 


### CRITICAL ANALYSIS

OF THE

### RECENT PUBLICATIONS

ON THE DIFFERENT BRANCHES OF

### PHYSIC, SURGERY, & MEDICAL PHILOSOPHY.

---

*A Fourth Dissertation on Fever, containing the History of, and Remedies to be employed in, irregular intermitting Fevers; by* GEORGE FORDYCE, M.D. F.R.S. &c. &c. *London*, 1802, 8vo. pp. 112.

WE have again to begin our Monthly Analyſis by a poſthumous work, the concluding labours of a veteran in the profeſſion of Phyſic, highly eminent for his ſtrength of mind and originality of thinking.

We cannot but conſider it as a fortunate circumſtance that the ingenious author was able to complete the Treatiſe before us, as it well concludes the Series of Eſſays of Fever, which he had ſucceſſively given to the public, if not by exhauſting the ideas which he might have to impart to his readers, at leaſt by carrying him beyond the limits of his perſonal experience; of an experience corrected by ſound ſenſe and acute obſervation, which gave ſo much value to his former Eſſays.

The ſubject of the preſent Diſſertation is the irregular intermitting Fever. The author begins with an explanation of the type of the ſeveral intermittents, and ſhews how, from occaſional circumſtances, the return of the exacerbation may be either haſtened or protracted, forming the *anticipating* or *retarding* intermittent. This accidental irregularity again may be repeated for ſeveral times ſucceſſively, till ſo many hours are either gained or loſt that the type may be changed, and a quotidian may be thus protracted, or a quartan anticipated, into a tertian. The indiſpoſition of a paroxyſm to return in the night time, very materially contributes to this total change of type. After ſeveral remarks on the type of Fevers, the author then proceeds to enumerate the irregularities and accidents which occaſion intermittents to quit the ſafe courſe of a regular tertian, and to become more dangerous in conſequence. The firſt of theſe accidents which he mentions, is a prolongation of the paroxyſm; and this is particularly liable to happen when inflammatory fever, or, as the author would term it, *general inflammation* is preſent. This diſeaſe, which is more commonly known by the term *inflammatory diatheſis*, is peculiarly characteriſed by hardneſs hardnefs of pulfe; and the mention of this term ferves to launch the author into a digreffion concerning this phenomenon, not irrelevant indeed to the fubject of Fever, but in which he introduces feveral of thofe obfervations concerning the coagulation of the blood, and the operation of the vital principle, which muft be familiar to all who have perufed the former works of this ingenious writer.

The Author then takes fome pains to prove, (what is not often doubted) that there really is fuch a thing as hardnefs of the pulfe; though Dr. William Hunter, as he informs us, conftantly denied that he could feel any fuch diftinction in the pulfation of the artery. This fenfation of hardnefs, the Author proceeds to obferve, is conftantly connected with a difpofition to flower coagulation in the blood than in time of perfect health; and hence he takes occafion to introduce the explanation of the appearance of the buff coat of inflamed blood; the circumftances of the fize of the orifice in venæfection; the form of the bafon in which the blood is received, &c. which are detailed with fufficient clearnefs, but unaccompanied with any original or very important obfervation. The fum and fubftance, therefore, of thefe remarks are contained in the following claufe:—"The Author further affirms, that when the pulfe has a feel of hardnefs, the blood taken from a vein is longer coagulating than when no hardnefs is to be felt; and that therefore there is actually a fenfation of hardnefs to be felt in the pulfe, in contradiction to all the other fenfations, viz. That the action of the arteries which gives this fenfation, alfo produces a difpofition to make the blood remain longer fluid after flowing from a vein into a bafon under the fame circumftances."

The reader might be at fome lofs what to expect after the following exordium, which begins the fucceeding paragraph:—"It is to be obferved, that every thing on this earth follows by gradation. The twilight which begins in the morning, gradually acquires meridian fplendour, and gradually finks from its meridian fplendour into the fhades of night. There is a gradation of animals from human beings, until they defcend through quadrupeds, fifh, and reptiles, and come down at laft to corals, fponges, and mufhrooms, until it is difficult to fay which is animal or vegetable.—*Such* gradations are there alfo in the feels of the pulfations of the arteries."

The fenfation of obftruction in the pulfe is next noticed, which the Author affirms, differs effentially from that of hardnefs, and derives its origin from a different affection of the pulfe, for the feel of obftruction may be very great; and yet the blood drawn from a vein at this time will coagulate immediately, and confequently not exhibit the buff coat.

It is certainly very difficult to exprefs by words that delicacy of feeling, that nicety of *tact*, which is only to be acquired by long experience in organs originally fenfible; but we may be allowed to obferve, that to fulfil any ufeful purpofe, there ought to be fome immediate and decided criterion between the feel of *hardness* and *obstruction*, independant of the fubfequent teft of the fpeedy coa-

 gulability gulability of the blood, ſince the object of this mode of examination of the ſtate of the circulation is to determine whether or not venæſection ought to be employed.

The Author then applies his favourite theory, concerning the cauſe of the coagulation of the blood, in the following words:—"The coagulable lymph, in conſequence of an animal's being alive, remains fluid; the mucilaginous part of it remains diſſolved in water. In the blood-veſſels of dead animals the mucilaginous part ſeparates from the water with which it is combined; it is therefore the life of the blood-veſſels which keeps the mucilaginous part diſſolved in the water, and conſequently the coagulable lymph fluid." From hence it is a legitimate inference, than when the blood is longer than uſual in coagulating, ſo as to form the buff coat, there muſt have been a ſuperior action of the living power in the arterial ſyſtem; and that, as hardneſs of pulſe indicates that ſtate of blood in which the buff coat would be formed after venæſection, it hence indicates equally the preſence of ſuperior action of the living power.

"This ſuperior action, (to return to the ſubject of intermitting Fever) when preſent, prolongs the time of each paroxyſm, and ſometimes proves fatal; perhaps by a ſudden affection of the brain. It alſo is liable, not only to prolong the paroxyſm, but to prevent the criſis being perfect; and thus the diſeaſe will have the appearance of a continued Fever, only with exacerbations at the times of the regular paroxyſm. This was called by the Greeks, Hemitriteon; and by Celſus, Semitertain."

The Author then proceeds to ſhew the effects of this continued inflammatory diatheſis on the quotidian and quartan types of Intermittents. The ſole remedy which he recommends in ſuch a caſe, is blood-letting; and he takes occaſion to introduce here a very clear and ſatisfactory view of the mechanical effect of this operation upon the arterial ſyſtem when under inflammatory action. Some obſervations on the employment of the auxiliary remedies of ipecacuanha, antimony, and cinchona, conclude the account of the irregular Intermittents in temperate climates.

From theſe the Author proceeds to the dreadful irregular ſemitertians, which form the direſt calamities in hot climates to the natives of more temperate regions. The infectious nature of the irregular intermittents of hot climates, has been a point of very warm controverſy; and the Author does not pretend to give a decided opinion on the ſubject, but rather inclines to the domeſtic origin of theſe diſeaſes during the heat of ſummer, and the conſequent putrefaction of animal and vegetable matter, (as in the inſtance of the memorable yellow Fever of Philadelphia) and ſuppoſes, that though all Fevers are, to a degree, infectious, this is not ſo in any very conſiderable degree. To theſe ſpeculations he adds a clear and accurate ſketch, or general deſcription, of the leading ſymptoms of the irregular Intermittent of hot climates, which he gives, not from actual obſervation, but from written accounts, counts, and from thofe given to him by medical practitioners, who themfelves have feen much of the difeafe, or have fuffered under it in their own perfons. For the medical treatment the Author takes upon him to condemn *all* the violent means that have been employed by the phyficians who have actually had the difeafe under their care; and in this fweeping condemnation he includes bleeding, purging, the ufe of mercury, and cold ablution; and recommends tartarized antimony, if the ftomach will bear it; cinchona, if any crifis comes on; but feems to be principally difpofed to leave the whole to Nature.

In the later part of this Treatife the Author returns to the irregularities that take place in the intermittents of temperate climates, the moft formidable of which are peripneumonic affections of the lungs; a propulfion of blood from the exterior parts to fome of the vifcera, particularly the fpleen and liver; tumours, apparently fchirrous, forming contiguous to the hepatic duct; jaundice; afcites; œdema; and fometimes diarrhœa. For the particular obfervations relative to the treatment of thefe fymptoms we muft refer the Reader to the Treatife itfelf; we fhall only add, that of the metallic preparations of arfenic, zinc, copper, and iron, which have ufually been employed in fimilar cafes, the Author has never fucceeded in enabling the conftitution to bear a full trial of the former; and of the three latter he fpeaks with a very guarded confidence.

---

*The Edinburgh School of Medicine, containing the preliminary or fundamental Branches of professional Education, viz. Anatomy, Medical Chemistry, and Botany; by* WILLIAM NISBET, M. D. *in four Volumes 8vo.* 1802.

THE medical world have given a favourable reception to the elementary work publifhed by the Author fome time fince, and entitled *the Clinical Guide.* The object of the prefent volumes is to form a fet of fchool books for the Student of Medicine, by exhibiting in a compendious view the principal facts relative to the elementary ftudies of Anatomy, Botany, and Chemiftry (the two latter confidered principally in a medical view) whereby the ideas of the Student may be directed, and the leading facts recalled to his memory. The arrangement which the Author follows is ftrictly that which is purfued by the Profeffors of Edinburgh; and this gives to thefe volumes a double advantage, to the Students of that illuftrious Seminary, of being able to employ this Compendium as a kind of text book; and to other readers, that of viewing in a concise form the arranged method which has appeared the moft eligible to fuch teachers as a Monro, a Black, and a Rutherford.

In a work which is profeffedly a Compendium, all that can be expected is clearnefs, accuracy, and a dextrous *sifting* of the whole mafs of materials, fo as to reject the digreffional and illuftrative part, and leave only that which is abfolutely effential to the fubject. We think that the Author has very well confidered the relative importance of the matter before him, by the fpace allotted to each. The two firft volumes are devoted to Anatomy, the third to to Chemiſtry, and the fourth to Botany; and the quantity of matter included in each is ſufficient for a pretty full ſketch of the different ſubjects. The moſt pains appear to us to have been beſtowed on the anatomical part, and in it the Author introduces occaſionally the outline of certain phyſiological diſcuſſions, which have received particular attention in the ſchool from which he has taken this compilation. The arrangement of the anatomical part is ſomewhat complicated, but, perhaps, not leſs inſtructive on that account; it attempts to include the conveniences of a diviſion into ſpecies, and one founded on the union of diſſimilar and complicated organs which perform the ſeveral functions. A ſketch of Morbid Anatomy is ſubjoined, ſelected principally from the valuable work of Dr. Baillie, to whom one of the volumes is dedicated. The whole of this part is executed with conſiderable ſkill and fidelity, and a few general plates are introduced, which are as illuſtrative as anatomical plates of this diminutive ſize can be expected to prove. We think, however, that a little more might have been done in what may be called the mechanical arrangement, in aſſiſting the eye of the reader: For inſtance, only two running titles are introduced; the firſt, "Oſteology," and all the remainder "Demonſtration and Diſſection," which laſt includes the whole of the ſoft parts, the muſcles, veſſels of every kind, nerves, viſcera, &c. A few of the names are tranſlated from the common Latin form into Engliſh; which exhibits rather a groteſque aſſemblage, though certainly not more abſurd than the original; ſo, we have the the terms, *sacred bone, rump bone, nameless bones, boot-like bones,* &c.; but, a reform of anatomical nomenclature is too gigantic an undertaking to be brought about by ſuch attempts.

The third volume includes the Chemiſtry, which, by the help of 700 pages of a cloſer type than the reſt, forms a compendium of conſiderable ſize, ample enough for all the neceſſary objects to the Medical Student embraced in this ſcience. The elementary treatiſes on Chemiſtry are ſo numerous that it would be hardly poſſible, with common induſtry, to avoid giving a compendium which ſhould contain abundance of valuable matter. It is principally in the ſelection and arrangement that the merit of the compiler conſiſts. We cannot find much to commend in either of theſe particulars, and we might point out ſeveral errors of ſome conſiderable magnitude. The order of general arrangement is Airs, Waters, Inflammables, Salts, Earths, and Metals; an order which it requires much knowledge in the writer to follow, conſiſtently with modern diſcoveries, without entangling and bewildering himſelf in a multiplicity of anomalies and repetitions. The whole Chemiſtry of Vegetable and Animal Matter follows by way of Supplement, and is diſpatched in very few pages; but for the Medical Student more attention might well have been beſtowed on them. A tranſlation of the London and Edinburgh Pharmacopeias is ſubjoined as the part peculiarly devoted to Pharmacy.

The fourth volume contains the Botany, a ſcience perhaps hardly ſufficiently attended to in medical education, but for the purſuit of which which more opportunities are wanted than generally fall to the lot of young men; ſince the conveniences afforded by large towns in the other branches of ſcience ſo ſtrongly recommend them as the ſeats of Schools and Univerſities. About half the preſent volume is occupied with a full and clear account of the ſyſtematical part of Botany and the Linnean arrangement: in the remainder the Author gives a ſuccinct account of particular articles employed in the Materia Medica, derived from the vegetable kingdom, in which the acknowledged authorities are followed with ſufficient exactneſs.

The errors of typography are numerous in theſe volumes, and particularly ſo in thoſe parts in which ſcientific names occur, a circumſtance of conſequence in an elementary book, as the Tyro may not be always able to correct them; and as the blame of ſuch numerous errors cannot with any fairneſs be thrown upon the ſhoulders of the preſsman or compoſitor, it muſt revert to the author.

---

*References to the Bones, for the Use of Anatomical Schools; by* Benjamin Simons, M. D. Edinburgh, 1801, pp. 131.

To underſtand the intention of this ſmall work, we muſt give the author's introduction. "Theſe References were compoſed for the uſe of an Anatomical School the author intends to inſtitute, and as a Syllabus to that department of a Courſe of Lectures which includes the bones. The plan is, to ſelect a ſet of the beſt marked bones, to annex to the proceſſes, foramina, &c. the numbers prefixed to their reſpective references: Thus labelled they are to be delivered to the ſtudent."

The ſmall publication before us is, therefore, ſuch a deſcription of the bones as it would be neceſſary for the ſtudent to retain in his memory; one in which all the parts worthy of note are diſtinctly enumerated; and might with little variation be converted into ſo many queſtions, whenever the teacher wiſhed to examine his pupil. It will be ſufficient to obſerve that they are accurate without being prolix; and any method which will aſſiſt the memory in the *Grammar* of Anatomy deſerves praiſe. The following will ſerve as an example.

"Inferior Spongy Bone.

Hanging low in the noſe, poſteriorly from the naſal lamella of the palate bone, and anteriorly from the ſuperior maxillary bone.

No. 1. Concave ſurface, facing the antrum.

2. Convex ſurface towards the ſeptum naſi.

3. Poſterior proceſs extended over a part of the antrum.

4. Anterior proceſs, connected to the os unguis, and ſuperior maxillary bone, ſo as to aſſiſt in forming the canal for the lacrymal duct.

The concave ſurface does not extend down to the inferior edge of the bone. The ſide of the inferior edge oppoſite the antrum is convex; and that to the ſeptum naſi, if not concave, is flat."

MEDICAL

